# Recent advances of additively manufactured noninvasive kinematic biosensors

**DOI:** 10.3389/fbioe.2023.1303004

**Published:** 2023-11-17

**Authors:** Jeonghoon Lee, Sangmin Park, Jaehoon Lee, Namjung Kim, Min Ku Kim

**Affiliations:** ^1^ Department of Mechanical Convergence Engineering, Hanyang University, Seoul, Republic of Korea; ^2^ Department of Mechanical Engineering, Gachon University, Seongnam, Republic of Korea; ^3^ School of Mechanical Engineering, Hanyang University, Seoul, Republic of Korea

**Keywords:** noninvasive biosensors, additive manufacturing, 3D printing, kinematic sensors, low-cost fabrication

## Abstract

The necessity of reliable measurement data assessment in the realm of human life has experienced exponential growth due to its extensive utilization in health monitoring, rehabilitation, surgery, and long-term treatment. As a result, the significance of kinematic biosensors has substantially increased across various domains, including wearable devices, human-machine interaction, and bioengineering. Traditionally, the fabrication of skin-mounted biosensors involved complex and costly processes such as lithography and deposition, which required extensive preparation. However, the advent of additive manufacturing has revolutionized biosensor production by facilitating customized manufacturing, expedited processes, and streamlined fabrication. AM technology enables the development of highly sensitive biosensors capable of measuring a wide range of kinematic signals while maintaining a low-cost aspect. This paper provides a comprehensive overview of state-of-the-art noninvasive kinematic biosensors created using diverse AM technologies. The detailed development process and the specifics of different types of kinematic biosensors are also discussed. Unlike previous review articles that primarily focused on the applications of additively manufactured sensors based on their sensing data, this article adopts a unique approach by categorizing and describing their applications according to their sensing frequencies. Although AM technology has opened new possibilities for biosensor fabrication, the field still faces several challenges that need to be addressed. Consequently, this paper also outlines these challenges and provides an overview of future applications in the field. This review article offers researchers in academia and industry a comprehensive overview of the innovative opportunities presented by kinematic biosensors fabricated through additive manufacturing technologies.

## 1 Introduction

The rapid evolution of wearable technology has led to a new era of personalized health monitoring and human-computer interaction. Among various groundbreaking innovations in this domain, wearable biosensors have emerged as a pivotal advancement, offering a unique blend of convenience, accuracy, and versatility. Kinematic biosensors, a subset of wearable biosensors, specialize in converting biological signals related to human motion into measurable electrical signals. As the demand for dependable measurement data in areas such as health monitoring, rehabilitation, surgery, and long-term treatment continues to grow, the significance of kinematic biosensors has been substantially increased (Reisner et al., 2007; Bourouis et al., 2011; Garcia et al., 2022; Ghadi et al., 2022; Tang et al., 2022). In contrast to chemical data sampling methods like blood draws, mechanical and electrical sensing enables the continuous and non-invasive monitoring of valuable biological signals, such as pulses, heart rates, and electrophysiology, for tracking organ and muscle activity without disrupting individuals’ daily routines (Kaisti et al., 2019; Baluta et al., 2022; Zheng et al., 2022). Recent developments have introduced various commercial wearables, including smartwatches, wristbands, and skin-mounted devices, which can provide electrical data related to various human motions, but they have encountered challenges related to suboptimal skin contact and confined measuring locations, resulting in reduced signal-to-noise ratios and limiting user mobility, respectively (Shakeriaski and Ghodrat, 2022; Stuart et al., 2022; Verma et al., 2022). The emergence of skin-interfaced devices offers a promising solution to the aforementioned challenges, enabling direct and conformal contact with the skin, even during significant mechanical deformation (Zhu P. et al., 2022). These innovative skin-interfaced biosensors are designed to seamlessly adhere to the skin and are able to collect physical, physiological, and biochemical signals emitted from the body. Their thin, soft, and multifunctional nature positions them as an ideal platform for personalized healthcare devices. Moreover, the advancement of additive manufacturing (AM) technologies has catalyzed a disruptive shift in the realm of skin-interfaced biosensors, transitioning from two-dimensional thin film formfactor to three-dimensional conformal structures (Ali et al., 2022). Recent AM technology offers the capability of fabricating complex geometries in high spatial resolution, incorporating vast freedom in material selections (metals, polymers, ceramics, and multi-material composites) and composite structures (Praveena et al., 2022). Therefore, in the realm of skin-interfaced biosensors, AM technology serves as a potent tool for the designing and fabricating of multifunctional devices, offering the ability to customize intricate, tunable, and cost-effective three-dimensional structures at various scales from micro to macro.

This article aims to provide an overview of recent research advancements in additively manufactured kinematic biosensors. A foundational understanding of AM techniques is essential to tailor these biosensors to their operational requirements. Consequently, the article is structured as follows: “Diverse AM Technologies” offers an in-depth exploration of fundamental AM techniques, elucidating their relevance to kinematic biosensors. “Types of Kinematic Sensors” outlines three distinct mechanisms of kinematic biosensors—namely, resistive, capacitive, and piezoelectric sensors. In “Applications,” the state-of-the-art additively manufactured kinematic biosensors are classified based on working frequency ranges (high, mid, and low frequencies), with a comprehensive examination of their utility in specific applications. Finally, the “Conclusion” section summarizes key findings and highlights prevailing challenges within this domain.

## 2 Diverse additive manufacturing technologies

**FIGURE 1:**
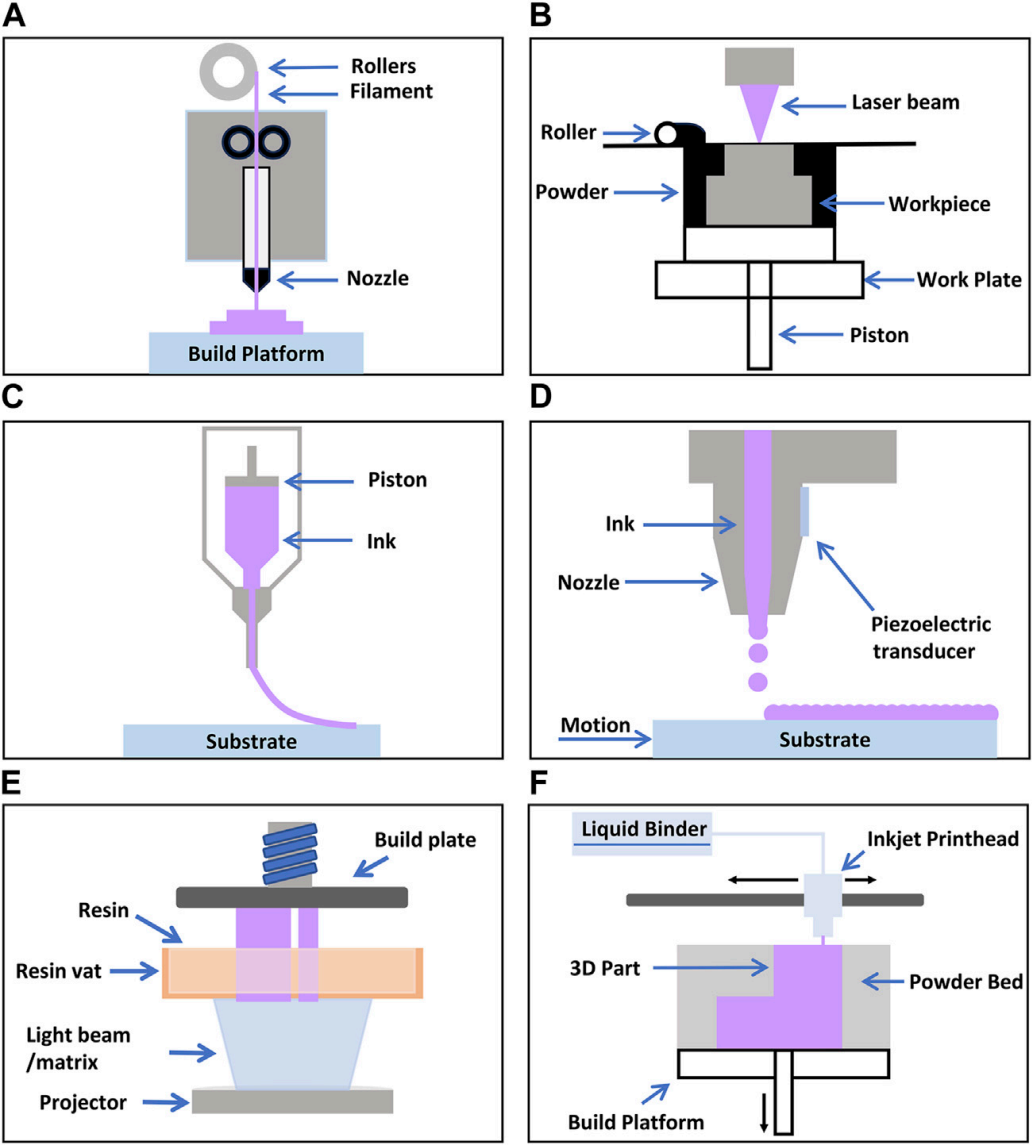
Schematic illustrations of various 3D Printing Processes **(A)** FDM (Mwema and Akinlabi, 2020) **(B)** SLS (Negi et al., 2014) **(C)** DIW (Liu et al., 2019) **(D)** IP (Xu et al., 2007) **(E)** DLP (Šafka Jiří et al., 2020) **(F)** Binderjet (Nikhil A et al.).

This section provides a brief overview of four representative additive manufacturing technologies: fused deposition modeling, selective laser sintering, direct ink writing, and inkjet printing, elucidating their relevance to biosensors. In addition, this section briefly touches on other additive manufacturing technologies such as digital light processing, and binder jet 3D printing. Figure 1 illustrates the schematics of these technologies.

### 2.1 Fused deposition modeling (FDM)

Fused Deposition Modeling (FDM) is a layer-by-layer manufacturing process in which a printer head supplies a thin plastic filament to the machine, which is then extruded through a material nozzle (Figure 1A) (Mwema and Akinlabi, 2020). Materials commonly used in this process include polycarbonate (PC), acrylonitrile butadiene styrene (ABS), and polyphenylsulfone (PPSF) (Cevenini et al., 2016; Dev and Srivastava, 2019; Lee et al., 2020). The primary advantages of FDM include eliminating the need for chemical post-processing, the absence of resin curing, and relatively low machinery costs. However, a drawback is that the vertical (*z*-axis) resolution is lower compared to other layering processes (∼0.25 mm), necessitating post-processing for smooth surface finishes when required. Additionally, it is a time-consuming process for creating large and structurally complex parts. To mitigate this time-related disadvantage, two modes that control the density of the final product are available: the fully dense mode and the sparse mode. While these modes save time, they may also compromise the mechanical properties of the model.

#### 2.1.1 Relevance to biosensors

One of the key advantages of FDM is its lack of need for chemical post-processing (Silva et al., 2020). This characteristic can be highly beneficial in the context of biosensor fabrication. Biosensors must react rapidly within a biological environment and deliver accurate measurements. Therefore, minimizing or entirely avoiding post-processing steps during sensor fabrication is crucial. FDM enables the production of biosensors without the need for additional chemical treatments or refinement processes, thereby enhancing the sensitivity and reliability of biosensors. Moreover, FDM does not require resin curing and involves relatively affordable machinery costs. Consequently, FDM can be utilized for large-scale production or customized manufacturing of biosensors. Since biosensors often require diverse designs and shapes, FDM can contribute to effectively realizing this diversity.

### 2.2 Selective laser sintering (SLS)

Using carbon dioxide laser beams, SLS is a three-dimensional printing process that fuses or sinters powder materials (Figure 1B) (Awad et al., 2020). In this process, the chamber is heated to the melting point of the material. The laser fuses the powder at the specific location of the model. SLS can accommodate a range of materials, including plastics, metals, metal-metal composites, metal-polymers, and metal-ceramics. Leveraging these advantages, SLS offers a wide spectrum of useable materials, and unused powders can be recycled. However, a drawback of the SLS process lies in the potential limitation of accuracy based on the particle size of the materials used. Furthermore, SLS requires execution in an inert gas environment to prevent oxidation and necessitates a consistent temperature near the melting point during the process. Considering these factors, SLS should be executed under appropriate conditions.

#### 2.2.1 Relevance to biosensors

One of the primary merits of SLS is its versatility in material fabrication. Biosensors require precise detection of specific biological signals or chemicals, often necessitating specialized materials (Muñoz and Pumera, 2020). SLS enables the creation of models using a variety of materials, from plastics to metals, allowing for optimal material selection to match the requirements of biosensors. Additionally, SLS provides high precision and durability, contributing to accurate measurements and long-term stability of biosensors. Given the importance of real-time and prolonged data collection for biosensors, sensors produced via SLS can support reliable monitoring by delivering accurate measurement results and extended lifespans. Furthermore, SLS possesses the capability to fabricate intricate shapes and designs. As biosensors are often intimately connected to biological entities, flexibility in design is essential for comfortable and effective wear. SLS can create models with unrestricted forms and structures, maximizing the wearability and efficiency of biosensors (Zhang et al., 2023).

### 2.3 Direct ink writing (DIW)

DIW, or Direct Ink Writing, is a three-dimensional process technology that precisely deposits ink or paste layer by layer to create 3D structures (Figure 1C). This process shares similarities with the 3D extrusion process of FDM; however, the distinguishing feature of DIW lies in the rheological properties of the ink, which, rather than drying or solidifying, maintain and shape the object’s form and structure as it exits the nozzle. DIW exhibits the capability to employ a wide array of materials in 3D printing production, ranging from ceramics, metal alloys, polymers, to biomaterials. Furthermore, DIW offers the advantage of relatively cost-effective production and the ability to construct complex shapes and structures without the need for additional molds. Nonetheless, DIW may encounter issues such as frequent nozzle clogging, necessitating a relatively low viscosity of the ink. Additionally, to prevent unwanted distortions, the ink viscosity should not be too low. Considering these drawbacks, DIW should be conducted with appropriate materials and conditions (Yang G. et al., 2021).

#### 2.3.1 Relevance to biosensors

A key advantage of DIW is the capacity to fabricate intricate structures with high flexibility (Gubanova et al., 2017). Biosensors often require close integration with biological entities and demand designs tailored to individual biological characteristics. DIW simplifies the creation of models with versatile forms and structures, enabling the realization of designs that satisfy the specific requirements of biosensors. Furthermore, DIW can utilize various materials. In the context of biosensors, materials with specific chemical or physical properties are frequently necessary (Erdem et al., 2023). DIW allows for the fabrication of models using diverse types of inks or substances, facilitating the selection of optimal materials aligned with the purpose and demands of biosensors (Ouyang et al., 2022).

### 2.4 Inkjet printing (IP)

IP is a technological process that utilizes inkjet printers to deposit precise ink onto the surface of an object, creating 2D or 3D structures through layer-by-layer stacking (Figure 1D). This process resembles the typical inkjet printing used for images or text on flat surfaces (Daly et al., 2015); however, in IP, the ink, which acts as the DIW material, maintains and constructs the shape and structure of the object as it exits the nozzle due to its rheological properties. IP operates by repetitively depositing ink to stack layers and create multi-layered structures. The distinct advantage of IP lies in its ability to utilize diverse materials for printing. This versatility extends to ceramics, polymers, organic and inorganic substances, and electronic materials, among others, making it applicable across various fields, notably in the realms of tissue engineering and electronic device industries (Wang et al., 2022). Key strengths of IP include providing high accuracy and resolution, enabling the cost-effective fabrication of intricate shapes and designs. Furthermore, its non-contact operation minimizes damage to the object’s surface. However, IP may have relatively slower speeds and can be time-consuming for larger areas. Moreover, the print quality and accuracy can be influenced by the characteristics of the ink, especially when dealing with smooth surfaces or precise boundaries between various materials. To overcome such limitations, appropriate inks and conditions, such as physical, dynamical, and rheological characteristics of inks and printing environment should be carefully selected when performing IP.

#### 2.4.1 Relevance to biosensors

A primary advantage of IP is the versatility of using a wide range of materials and colors. Biosensors often require the detection of specific biological signals or chemicals in various ways, emphasizing the importance of flexibility in material and color usage during fabrication (Yakovlev et al., 2016). Inkjet Printing accommodates different types of inks and substances, allowing for the creation of models tailored to the specific demands of biosensors. Additionally, IP has the advantage of high-resolution printing (Zhang et al., 2014). As biosensors demand precise measurements, Inkjet Printing’s high resolution facilitates the fabrication of models that yield accurate measurement results, thereby enhancing the sensitivity and accuracy of biosensors. Consequently, IP can elevate the performance of biosensors. IP also stands out for its relatively straightforward equipment and cost-effective production. With biosensors often requiring either large-scale production or cost-efficient individual sensor fabrication, IP can efficiently meet these demands. However, it requires attention to ink drying and adhesive maintenance during the stacking process. Particularly when crafting sophisticated structures that can react to the small kinematic signals, careful design and fabrication are necessary, considering the influence of ink properties and stacking methods.

### 2.5 Other additive manufacturing technologies

Various alternative printing techniques have been proposed as valuable for the fabrication of 2D or 3D structures. In this context, we briefly provide a concise overview of other mechanisms, highlighting their applicability in the production of noninvasive kinematic biosensors.

DLP (Digital Light Processing) is a digital light processing technology that enables the creation of 2D or 3D structures using light (Lu et al., 2006; Choi et al., 2009). In this process, a laser beam or other light source is used to display digitally controlled patterns on a reflective mirror. These patterns are then employed to cure materials, forming the desired structure. This technology allows for the fabrication of precision components and models in diverse areas, including optical lenses, semiconductors, and dentistry applications. Notable strengths of DLP include its capacity to provide high precision and resolution, as well as relatively fast structure formation. Moreover, it can handle large areas at once, making it suitable for mass production. However, DLP typically relies on specific materials, and controlling the photopolymerization reaction for some substances can be challenging (Zhao Z. et al., 2020). Additionally, due to the cured structure serving as a stencil, there can be limitations within the internal structure. Consequently, careful consideration of materials and the manufacturing process is necessary when working with DLP.

Binder Jet 3D printing is one of the powder-based 3D printing technologies that involves precisely stacking solid powder materials in layers and binding them using a binding agent, creating a 3D object. Binder Jet technology can utilize various materials and finds applications in various fields, including metals, ceramics, plastics, and more (Ziaee and Crane, 2019). This capability to perform multi-material printing offers the advantage of maximizing the characteristics of each powder material. However, it is important to note that Binder Jetting may necessitate subsequent post-processing procedures and may lead to lead to unintended deformations, imposing limitations in terms of accuracy and resolution (Du et al., 2020). Furthermore, in terms of material strength and durability, Binder Jetting may exhibit certain limitations in comparison to conventional manufacturing methodologies. Consequently, careful evaluation of the suitability of Binder Jetting is essential, contingent upon the specific materials and applications under consideration.

## 3 Types of kinematic biosensors

**FIGURE 2:**
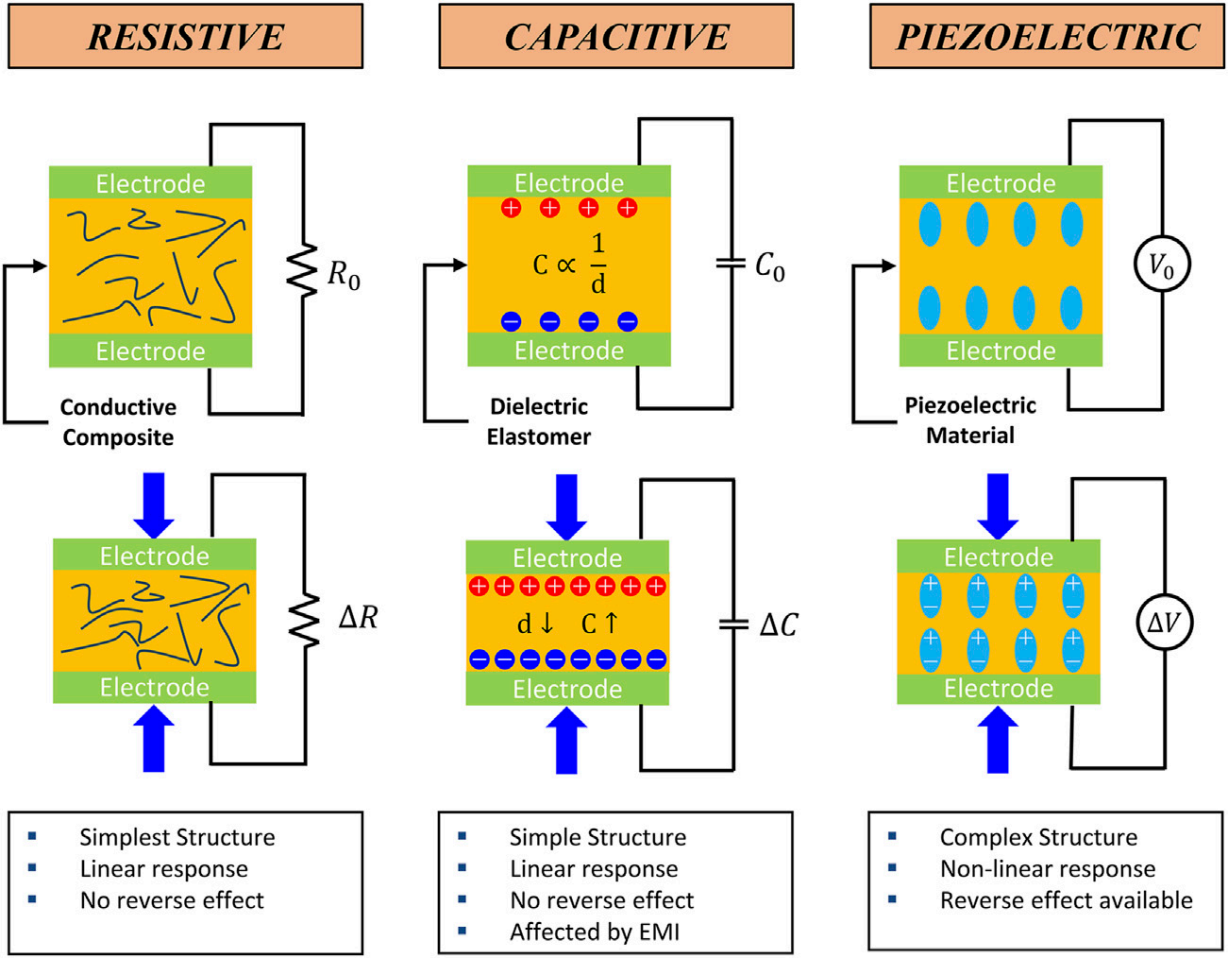
Schematical working principle of the typical kinematic sensors and their features (Lee et al., 2020; Osman and Lu., 2023).

A kinematic biosensor is a biosensor that converts mechanical bio-signals such as strain, pressure, and tactile stimuli into an electrical signal. Kinematic biosensors can be divided into several categories according to their sensing (transduction) mechanism. Among many mechanisms, resistive, capacitive, and piezoelectric sensors are most utilized due to their simple structure, ease of fabrication, and high fidelity. The working principle and features of each sensor type are described in Figure 2. In addition, triboelectric sensors are briefly discussed. Diverse methods exist to enhance sensor performance. Developing new materials or the innovative amalgamation of existing materials has notably contributed to refining sensor functionality, such as sensitivity, linearity, and mechanical adaptability. Significantly, additive manufacturing has emerged as a catalyst in advancing biosensor capabilities, primarily by enabling the fabrication of intricate sensor architectures. Beyond the scope of material selection, the diversification of sensor structures has yielded a cohort of exceptional high-performance sensors, thereby marking a substantial advancement in the field. A summary outlining the printing method, material composition, performance, and durability of the additively manufactured biosensors is presented in Table 1.

**TABLE 1:** Overview of the additively manufactured biosensors.

Working mechanism	3DP method	Printing materials	Sensitivity or gauge factor (GF)	Durability	References
Resistive	FDM	Acrylonitrile butadiene styrene (ABS) filament	GF 10	over ∼12 months	Davoodi et al. (2020)
	DIW	carbon black (CB)/polydimethylsiloxane (PDMS) composites	0.0048 kPa^-1^, GF 4.8	stable over 500 cycles	Zhu et al. (2022a)
	DLP	Hydrolysable scaffold (sacrificial mold)	0.111kPa^-1^	100 compression cycles (60% strain)	Peng et al. (2021)
	DIW	TPU, PDMS, Ecoflex, PI, TPU/CB/NaCl, TPU/Ag	5.54 kPa^-1^, GF 13.3	10,000 loading-unloading cycles	Wang et al. (2019)
	FDM	TPU	2.68MPa^-1^	1,000 loading cycles	Kim et al. (2023)
	DIW	Carbon Black/Ecoflex, Nano Silica/Ecoflex, Nano Silica/E650 ink	GF 13.4	500 cycles (10% strain)	Wang et al. (2021)
	SLA	The flexible photosensitive resin	419.622kPa^-1^	1,000 cycles (10 kPa pressure)	Xia et al. (2021)
	DIW	Conductive silver nanoparticle (AgNP)	0.48kPa^-1^	1,000 cycles (1.5 kPa pressure)	Lo et al. (2020)
	DIW	graphene/PDMS	GF 448	100 compression cycles (10% strain)	Huang et al. (2019)
	AJP	AgNPs	GF 9–10	N/A	Agarwala et al. (2018)
	FDM	TPE, TPU	GF 180	3 days	Guo et al. (2017)
	FDM	TPU filament, CNT/TPU filament	N/A	1,000 bending	Kim et al. (2017b)
	DLP	NaCl doped AG	GF 17	500 continuous cycles	Su et al. (2020)
	hybrid DLP	UV-curable multiwalled carbon nanotubes/elastomer (MWCNT/EA) composite	15.04MPa^-1^	6,000 cycles (5% strain)	Xiao et al. (2023)
Capacitive	DLP	ionic conductive hydrogel, water-dilutable polyurethane acrylate (WPUA)	0.25–0.61kPa^-1^	10,000 pressure cycles	Yin et al. (2019)
	DIW	PDMA-C18 hydrogel	0.45kPa^-1^	10 dynamic thermal sensing	Lei et al. (2017)
	DLP	MA/ChCl-AAm/ChCl type PDES precursor solution	8.92kPa^-1^	500 cycles (160 kPa pressure)	Wu et al. (2022)
	DLP	mixture of photocurable resin and MWCNTs	GF 38	10 cycles (2.5% strain)	Mu et al. (2017)
	FDM	ABS	N/A	N/A	Huang et al. (2017)
	AJP	AgNP ink	N/A	N/A	Rahman et al. (2016)
	FDM	carbon black thermoplastic polyurethane (PI–ETPU), thermoplastic polyurethane (TPU)	3.54%–8.67%	N/A	Loh et al. (2021)
	FDM	Commercial filament	0.583kPa^-1^	200 loading cycles (50 kPa pressure)	Zhao et al. (2023)
	DIW	mesoporous PDMS	0.0121–44.5kPa^-1^	N/A	Yang et al. (2021b)
Piezoelectric	DLP	micro Pb [(Mg1/3Nb2/3)0.1Zr0.45Ti0.45]O3 (PMN–PZT) ceramic–polymer composites with a silane coupling agent and dispersant	N/A	N/A	Chang et al. (2023)
	SLA	pristine BNNT and surface modified BNNT with photocurable resin	120 mV/(kPa?wt%)	N/A	Zhang et al. (2020)
	MIP-SL	BaTiO3 powder mixed with photocurable resin	N/A	N/A	Zeng et al. (2020)
	SLA	barium titanate nanoparticles into photoliable polymer solutions	N/A	N/A	Kim et al. (2014)
	SLA	surface functionalized PZT nanoparticle and photosensitive monomer	N/A	N/A	Cui et al. (2019)
	SEA-3DP	mixture of DMF, PVDF, DMSO	N/A	N/A	Bodkhe et al. (2018)
	FDM	PVDF filament	N/A	N/A	Kim et al. (2017a)
	DIW	PVDF nano micro fibers	N/A	N/A	Fuh et al. (2017)
	FDM	PVDF filament, Commercial conductive filament	N/A	N/A	Košir and Slavič (2022)

### 3.1 Resistive sensor

Resistive-type sensors find widespread utility across diverse domains, including biomechanics, wearable devices, and human-machine interfaces. They are the predominant category of sensors due to their simple configuration, facile manufacturing process, and high accuracy. Generally, a resistive sensor comprises a pair of electrodes encapsulating a conductive substance. The sandwiched material is functionalized with conductive properties by encompassing materials like carbon-based fillers (Wang et al., 2021; Xiao et al., 2023), metal fillers, metal-coated particles (Lo et al., 2020), conductive polymers (Liu et al., 2019), and hybrid fillers (Zhao C. et al., 2020). The fundamental principle underlying resistive sensors involves detecting kinematic stimuli through quantifying resistance changes when the mechanical stimulus is exerted. This alteration in resistance emerges from the conductive fibers and particles interposed within the composite structure. Compressing the conductive composite causes a reduction in the inter-fiber and inter-particle spacing, thereby augmenting the composite’s overall conductivity, and leading to a change in resistance. However, this resistance modulation is not uniform, exhibiting variability contingent upon various factors such as sensor geometries, material types, material composition, and other determinants. These factors impact the output signal linearity and influence other sensor attributes, such as sensing range and sensibility.

In this context, the role of additive manufacturing introduces key advantages. Through tailored fabrication, additive manufacturing substantially enhances the performance of resistive-type sensors. In contrast to conventional manufacturing methods characterized by arduous and costly processes, 3D printing facilitates the fabrication of complex sensor geometries in simplified processes. This advancement in fabrication technology markedly expands the design landscape of sensors, ensuring their efficacy and applicability in diverse domains.

**FIGURE 3:**
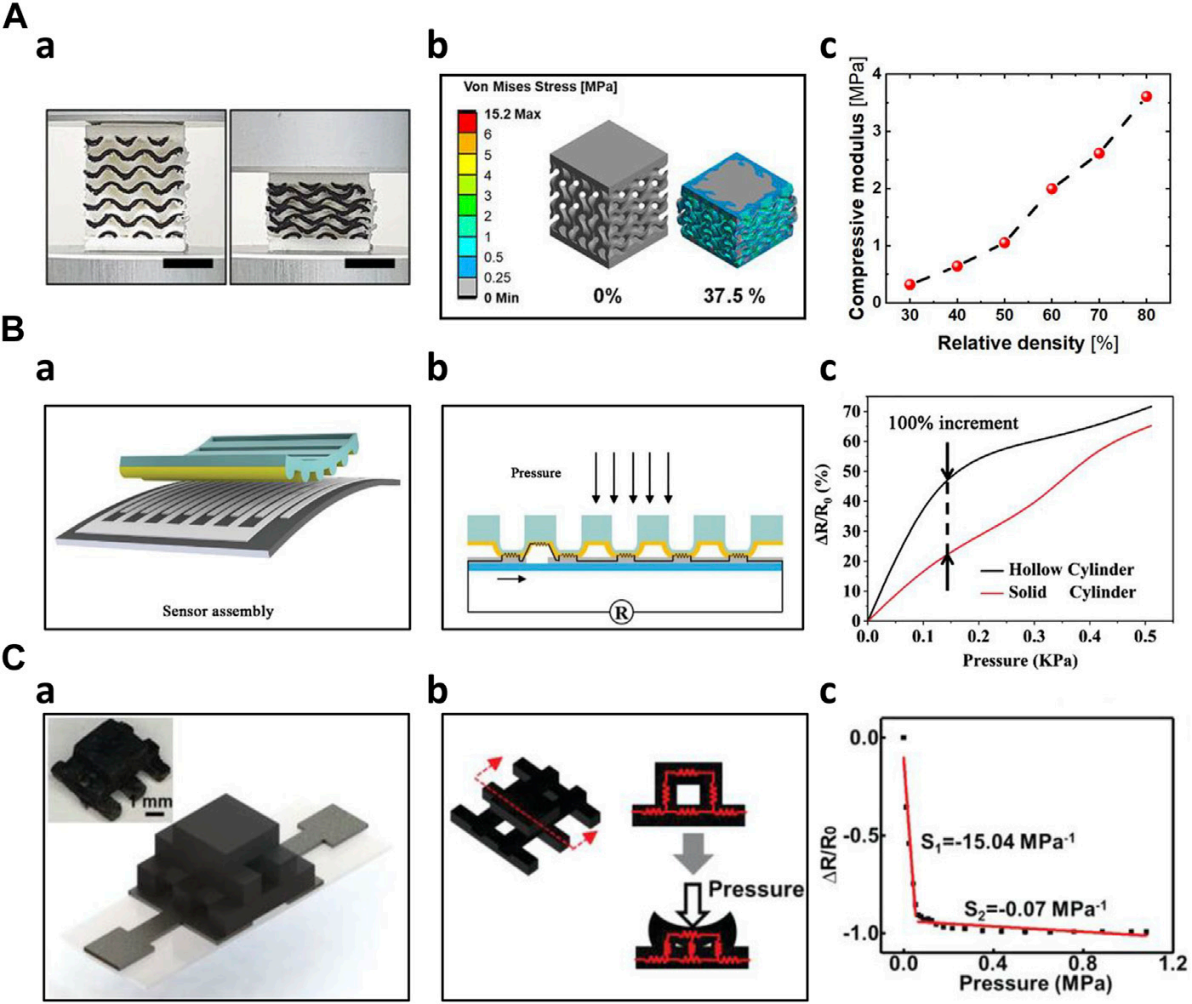
3D Printed Resistive Sensors **(A)** FDM printed porous gyroid pressure sensor (Kim et al., 2023) a) 3D-printed gyroid pressure sensor on 0%, 37.5% compression b) Stress distribution simulation of the gyroid pressure sensor on 0%, 37.5% compression c) Effective compressive modulus according to different relative densities **(B)** 3D printed hollow microstructure-based pressure sensor (Xia et al., 2021) a) Schematical image of the sensor assembly b) Sensing mechanism of the microstructure-based pressure sensor c) Comparison of the sensor performance of hollow cylinder and solid cylinder **(C)** DLP 3D printed sensor with woodpile configuration (Xiao et al., 2023) a) Schematic and optical image of the woodpile configuration sensor b) Sensing mechanism of the woodpile configuration sensor c) Sensing behavior of the woodpile configuration sensor.

Several researchers have demonstrated improvement in sensor performance by introducing a porous structure within the sensor. The adoption of a porous structure facilitated broad sensing capability due to its flexibility. Davoodi et al. fabricated a porous resistive sensor to improve its sensitivity using sacrificial template molding. The sacrificial mold was FDM 3D printed with ABS and then cast with silicone rubber. The ABS sacrificial mold was dissolved with acetone, leaving the silicone rubber behind. The resulting silicone rubber, either embedded with graphene, then the performance of the fabricated surface embedded graphene (SEG) sensors and surface deposited graphene (SDG) sensors were compared. Both sensors demonstrated pros and cons in sensitivity, durability, and biocompatibility (Davoodi et al., 2020). Inspired by human skin nature, Zhu et al. fabricated a flexible pressure sensor with a gradient porous structure. The skin-inspired gradient sensor showed a sensitivity of 4.8 Pa^−1^ over a wide sensing range (0–500 kPa) and reproducibility over 500 compression cycles without performance degradation. In addition, using FEA simulation, researchers compared and analyzed the resistive response of different porous structures (Zhu G. et al., 2022). Peng et al. fabricated a porous flexible strain sensor (PFSS) by casting polyurethane/carbon nanotube composites into 3D-printed sacrificial molds. The fabricated sensor showed a stretchability of 510% with an excellent recovery ability (10% of total strain loss after 100 compression loading cycles). The sensor also exhibited stable resistance measurement under 100 large strain applications (Peng et al., 2021). Wang et al. adopted a hierarchical porous structure with multi-modulus architecture to improve the sensitivity with increased sensing range. The porous structure efficiently distributed the stress, allowing a 7% resistance change over 50% strain. Furthermore, the sensor showed a high sensitivity of 5.54 kPa^-1^, and a large sensing range from 10 Pa to 800 kPa (Wang et al., 2019). Kim et al. directly printed porous gyroid structures using the FDM 3D printing method with TPU. The printed structure was then coated with CNTs to be used as a piezoresistive pressure sensor. Young’s modulus of the gyroid structured sensor could be controlled by changing the density of the gyroid structure. For instance, the sensor exhibited Young’s modulus of 0.32 MPa, and 3.61 MPa at 30% and 80% relative density, respectively. In addition, the fabricated sensor showed a linear response of up to 37% strain. Due to the programmable density of the sensor, it demonstrated a wide sensing range of up to 1.45 MPa and a high sensitivity of 2.68 MPa (Figure 3A) (Kim et al., 2023).

In addition to porous structures, other novel structures, such as auxetic, microcylinder, and serpentine-shaped structures, were fabricated by additive manufacturing technology. Combining DIW and Ink Spraying Technique (IST), Wang et al. fabricated an auxetic bilayer mesh strain sensor. This mesh-based structure sensor showed a high gauge factor of up to 13.4, 6.6 times larger than the non-structured flat sensor. The sensor also demonstrated a wide sensing range (*ε* > 20%) with high linearity (*R* ^2^ > 0.990) and good durability (more than 500 cycles) (Wang et al., 2021). Xia et al. used 3D printing to fabricate a hollow microcylinder structure to improve the performance of the pressure force under the compression force. As the pressure was applied, the microstructures induced the electrode microcrack effect, which improved its sensitivity. Under pressures below 100 Pa, the hollow microcylinder structured sensor showed a sensitivity of 419.622 kPa^-1^, two times higher than the solid type. Furthermore, the novel structured sensor showed a rapid response time of 30.76 m and a recovery time of 15.17 m (Figure 3B) (Xia et al., 2021). Lo et al. employed inkjet printing technology to produce serpentine-shaped pressure sensor patches designed for wearable applications. These patches featured a silver nanoparticle (AgNP) layer directly printed onto the polydimethylsiloxane (PDMS) layer, serving as electrodes. The top layer was covered with very high bond (VHB) tape. By detecting the strain induced by the pressure, the sensor was able to measure the pressure applied to the sensor surface (Lo et al., 2020). Huang et al. fabricated a three-dimensional graphene-PDMS (3DGP) by altering the filament diameter, interaxial angle, and interlayer space. By changing such parameters, the 3DGP structure could be used as a highly sensitive sensor with controllable sensor performance. These micro-structured sensors exhibited large gauge factor up to 448 at 30% strain. Furthermore, the 3DGP scaffold had negligible performance degradation even after 100 compressions under 10% strain (Huang et al., 2019). Utilizing the design flexibility of additive manufacturing, Agarwala et al. proposed diverse structures of aerosol-jet printed sensors and selected the ideal structures using commercially available simulations. Five different grid designs were proposed for improvement in gauge factor. Substrates were optimized for higher sensitivity. Prior to the actual manufacturing of the sensors, the addition of the simulation yielded higher efficiency of ideal sensor design (Agarwala et al., 2018). Guo et al. 3D printed a tactile sensor consisting of four independent layers. The base layer was printed in a flat, solid structure using silicone ink. On top of the base layer, a grid-structured electrode layer and a helical coil-shaped sensor layer were printed using different compositions of Ag/Silicone composite. The isolating layer and supporting layer were also 3D printed. Additive manufacturing technology enabled complex composite layered sensor fabrication with ease, which was not easily achieved with traditional manufacturing technologies (Guo et al., 2017). Using FDM 3D printing, Kim et al. fabricated a cubic cross-shaped force sensor to measure forces in three dimensions. The structure effectively distributed the forces onto each axis. They used functionalized nanocomposite filament consisting of CNT/TPU for the sensing parts and commercial TPU filament for the structural parts (Kim K. et al., 2017). Su et al. utilized a DLP 3D printing method to develop a hollow elastomer-shaper that is filled with NaCL@AG solution. The hollow part was designed as a combination of straight channel and helical channel, which exhibited superior sensing performances as a large strain sensor. The sensor demonstrated a gauge factor of 17 at 500% strain. In addition, the sensor was able to detect both bending and stretching motions (Su et al., 2020). Xiao et al. adopted a woodpile structure, which showed improved sensitivity nine times higher compared to the flat solid type of sensor. Using novel hybrid DLP-3D printing, an MWCNT/EA sensing unit was developed. Solid-type sensors exhibited a sensitivity of 1.62 MPa^-1^ under the pressure of 78.4 kPa. However, the woodpile structured sensor showed a sensitivity of 15.04 MPa under pressure of 64.44 kPa (Figure 3C) (Xiao et al., 2023).

### 3.2 Capacitive sensor

Numerous applications of capacitive sensors in biosensors include artificial skin (Mannsfeld et al., 2010), wearable electronics, and touch-screen devices. Notably, capacitive sensors are used in artificial skins like electronic or ionic skins due to their high sensitivity to small pressure changes. Electronic skins utilize electronic components, whereas ionic skins employ materials capable of conducting ions, each mimicking distinct aspects of human skin behavior. In contrast to resistive sensors, capacitive sensors offer the advantage of exhibiting linearity in their sensing behavior (Chortos et al., 2016). However, it is important to note that capacitive sensors can be susceptible to surrounding electromagnetic interference. Capacitive sensors are typically comprised of dielectric elastomers sandwiched between conductive electrodes. When external pressure or strain is applied the distance between the electrodes decreases, causing an increase in the capacitance of the dielectric. This change in capacitance makes it possible to sense and measure the applied pressure or strain.

**FIGURE 4:**
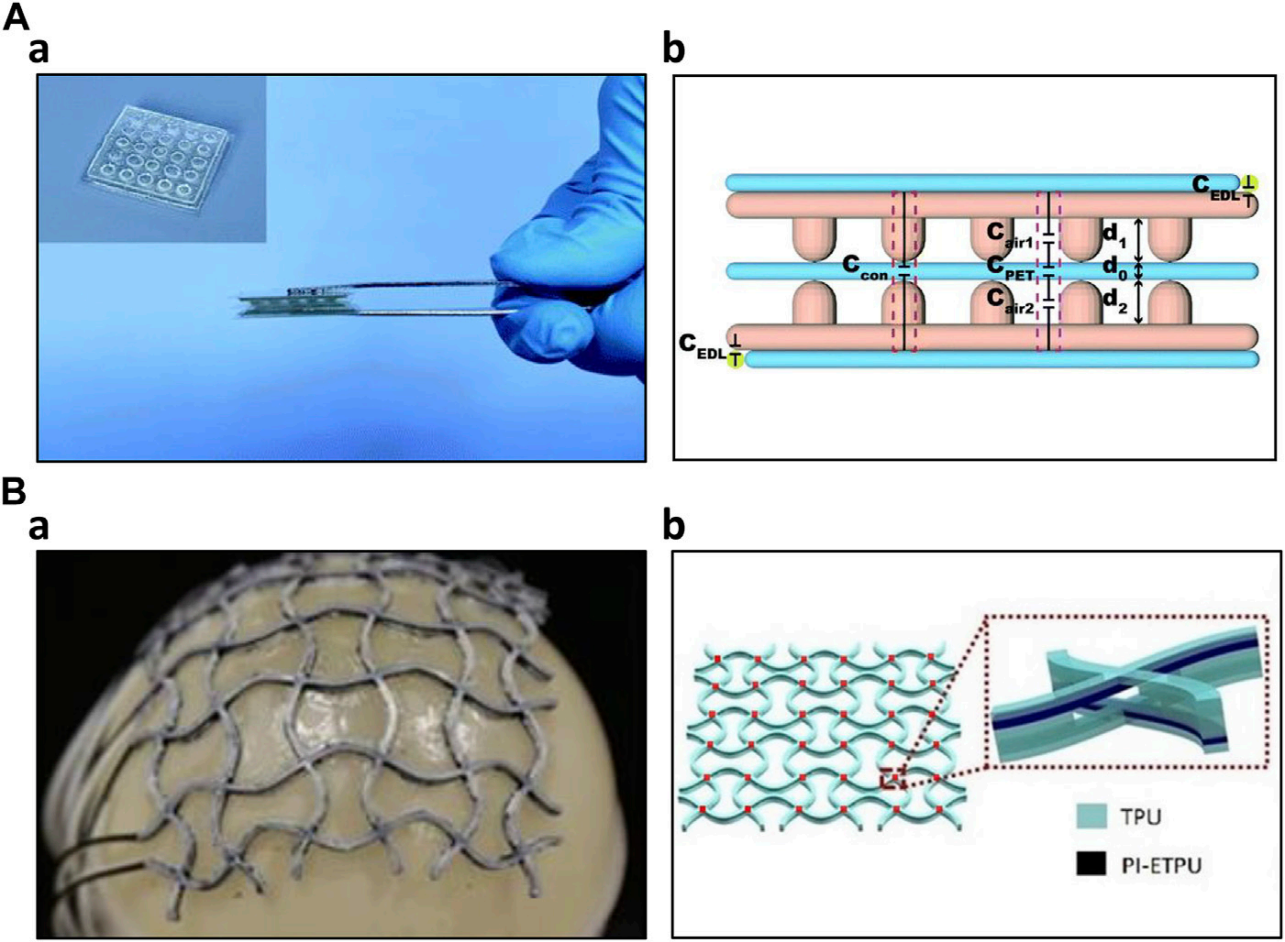
3D Printed Capacitive Sensors **(A)** 3D printed capacitive ionic skin (Wu et al., 2022) a) Optical image of the ionic skin b) Schematic structure of the capacitive ionic skin **(B)** 3D printed capacitive sensing array (Loh et al., 2021) a) Image of the sensing array conforming to the balloon surface b) 6 × 6 capacitive sensor array and its layer structure.

In the field of artificial skin fabrication, resistive and capacitive mechanisms have been widely employed due to their sensitivity. Capacitive sensors have gained significant attention due to their outstanding sensitivity, linearity, low hysteresis, low power consumption, temperature independence, frequency response, and long-term stability compared to resistive sensors. However, piezoelectric sensors are not widely adopted in this context because it cannot detect static pressure effectively. Additionally, the temperature susceptibility of piezoelectric sensors has further limited their usage in artificial skin applications (Chortos et al., 2016; Yin et al., 2019). Yin et al. developed a DLP dual-material printing method to fabricate ionic skins for elaborate pressure sensing. Two materials were alternatively printed to compose a capacitive sensing layer. Hydrogel served as electrodes, whereas water-dilutable polyurethane acrylate (WPUA) was a dielectric layer sandwiched between two electrodes. By printing microstructures, the ionic skin had five times higher sensitivity than the non-structured one. Furthermore, the printing process endowed the ionic skin eliminating signal drift and performance degradation for long-term use (Yin et al., 2019). Lei et al. created a multifunctional skin-like sensor by crafting a thermo-responsive double-network hydrogel through a micellar-copolymerization technique. This hydrogel exhibited the capability to manage its rheological properties, allowing for the 3D printing of microstructures with sub-millimeter precision. The application of additive manufacturing technology, in conjunction with the appropriate materials, improved the ionic skin’s sensitivity (Lei et al., 2017). Wu et al. have developed a highly sensitive solid-state capacitive ionic skin (SCIS) known for its stability even in harsh environmental conditions. They employed 3D printing to photopolymerize two polymerizable deep eutectic solvents (PDESs): acrylamide (AAm)/choline chloride (ChCl) and maleic acid (MA)/ChCl type PDESs, resulting in a random copolymer network. In summary, the utilization of additive manufacturing through UV light contributed to creation of a robust sensor with a remarkable sensitivity of 8.92 kPa^-1^ and a wide sensing range spanning from 50 Pa to 10 MPa. Furthermore, they demonstrated the microstructure achievable through this fabrication method (Figure 4A) (Wu et al., 2022).

Additive manufacturing technology also enhanced the capacitive sensor performance by allowing complex structures. Using additive manufacturing, Mu et al. produced various conductive structures, including helical coils, planar waves, and hollow truss structures. They optimized parameters such as the loading of multi-walled carbon nanotubes (MWCNT) and digital light processing (DLP) printing settings like layer thickness and light irradiation time. Notably, they showcased the potential applications of a hollow-structured capacitive sensor featuring network-shaped electrodes comprised of MWCNT nanocomposites. This sensor displayed a capacitance response correlated with nominal strain, reaching up to 70%, while maintaining reasonable repeatability and sensitivity (Mu et al., 2017). Huang et al. constructed a three-axial force sensor utilizing a hemispherical bump structure composed of PDMS fabricated through a 3D printing process. This hemispherical bump structure efficiently decomposes external forces in three-axis directions. Furthermore, the dielectric layer, consisting of an air layer and a PDMS layer, enhanced the dielectric coefficient (Huang et al., 2017). Rahman et al. produced interdigitated capacitive touch sensors employing aerosol jet printing (AJP) technology. This additive manufacturing process significantly lowered fabrication costs and mitigated chemical-related hazards. Through AJP printing, they achieved interdigitated electrodes with dimensions as small as 45 μm. Subsequent scanning electron microscope (SEM) and atomic force microscope (AFM) examinations demonstrated some variability in electrode thickness but minimal capacitance fluctuations, signifying that the fabrication process is sufficiently reliable for stable sensor operation (Rahman et al., 2016). To detect normal forces and stretching stimuli on curved surfaces, such as body joints, Loh et al. developed a 3D-printed metamaterial capacitive sensing array. This sensor array was created using multimaterial FDM 3D printing. It consisted of several layers, including two TPU insulation layers, two PI-ETPU electrode layers, and a TPU dielectric layer, printed in an auxetic shape. The vertices of the sensor array served as capacitive sensors. It is noteworthy that the sensor performance remained unaffected even when subjected to uniaxial stretching of up to 21.6%. Using this resulting sensor array, the researchers demonstrated its applicability in a soft universal jamming gripper and as a conformal force-sensing wearable on an elbow (Figure 4B) (Loh et al., 2021). Inspired by the structure of frog legs, Zhao et al. incorporated this design into the dielectric of their capacitive pressure sensor. They utilized 3D printing to create mold for the substrate and the frog-leg-like dielectric. After fabrication, the structure’s finer details were optimized through experimental studies and further evaluated using finite element analysis (FEA). This biomimetic structure effectively reduced the Young’s modulus of the entire dielectric while maintaining stability. Additionally, it significantly enhanced the sensor’s performance, offering reliable stability, rapid response (40 ms), and quick recovery (45 ms) at 1 kPa, along with ultrahigh sensitivity of 0.583 kPa^-1^, low detection limit of 0.5 Pa, and a wide sensing range spanning from 0 to 200 kPa. The capacitive sensor, inspired by nature and fabricated using the 3D printing process, demonstrated exceptional performance, making it suitable for a wide range of applications (Zhao et al., 2023). Yang et al. have developed a highly sensitive capacitive sensor using Direct Ink Writing (DIW) printing technology. They 3D printed the dielectric layer in a structured manner, referred to as the 'face-centered tetragonal (FCT)' structure. Mesoporous PDMS ink was alternately printed horizontally and vertically, creating an alternating layer. The resulting dielectric layer was then placed between electrodes of electroless copper-plated PI films. By controlling parameters such as spacing, porosity, and the specific stacking structure, the researchers determined that the optimal sensitivity was achieved with four layers of 260 μm diameter filaments and a spacing of 800 μm. This configuration exhibited a sensitivity of 1.23 kPa^-1^ within the sensing range of 0–10 kPa, with sufficient reproducibility (Yang W. et al., 2021).

### 3.3 Piezoelectric sensor

Piezoelectric sensors are broadly utilized in fields such as pressure sensors (Yang et al., 2020), self-powered sensors (Yuan et al., 2019), and ultrasonic devices (Akasheh et al., 2004; Chen et al., 2016; Zeng et al., 2020). Piezoelectric sensors make use of a variety of piezoelectric materials, such as quartz, lead zirconate titanate (PZT), barium titanate (BaTiO3), and polyvinylidene fluoride (PVDF). Moreover, these materials can be integrated into flexible polymers to enhance their mechanical properties. These sensors operate based on the intrinsic piezoelectric properties of these materials. The dipoles become charged when strain or pressure is applied, inducing electric potentials. The resulting voltage changes are used to measure kinematic data such as strain or pressure, allowing for precise sensing in various applications.

**FIGURE 5:**
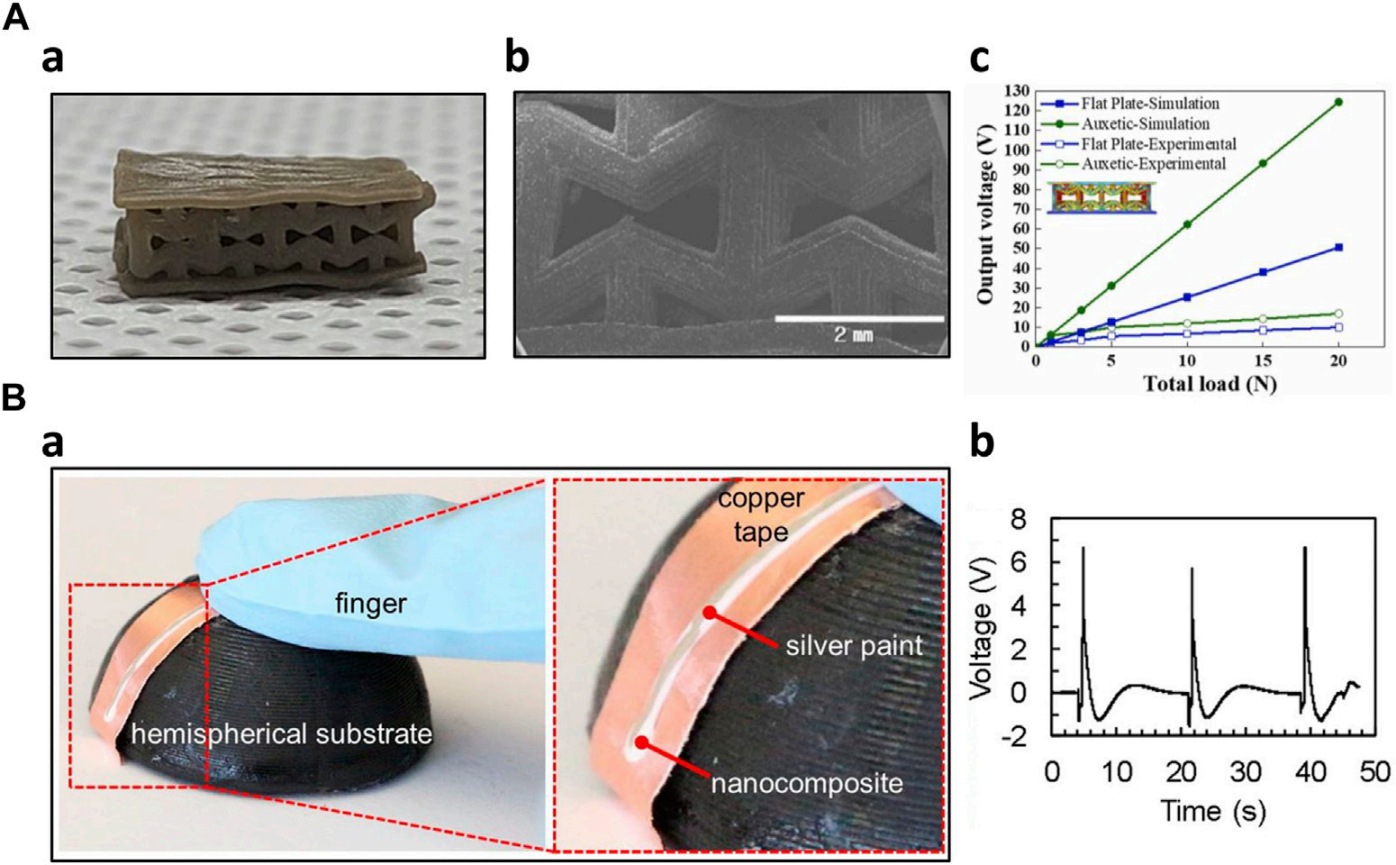
3D-printed piezoelectric sensors **(A)** Optimized auxetic tactile sensor (Chang et al., 2023) a) Optical image of the auxetic shaped sensor b) SEM image of the microstructure c) Piezoelectric output comparison of auxetic shaped sensor with flat plate sensor **(B)** Solvent-evaporation, electric poling assisted 3D printed piezoelectric sensor (Bodkhe et al., 2018) a) Fabricated conformal sensor b) Piezoelectric output to finger pressing.

Additive manufacturing has significantly enhanced sensor performance by enabling efficient and rapid fabrication of complex sensor structures. Chang et al. optimized both the formulation and structure of the piezoelectric composites. Initially, they optimized the formulation and fine-tuned printing parameters, including curing depth and excess width, for the 3D-printed composites. This optimization process resulted in enhanced sensing performance coupled with improved mechanical robustness. Utilizing digital light processing (DLP) 3D printing, the researchers printed various auxetic structures and compared their performance. They employed finite element method (FEM) simulations to predict the most promising structure with the highest piezoelectric voltage output, which was then experimentally validated. Remarkably, the auxetic structures demonstrated three times higher output voltage than flat structures (Figure 5A) (Chang et al., 2023). Zhang et al. engineered piezoelectric nanocomposites with improved performance by incorporating functionalized additives and microstructuring the composites through 3D printing. This was achieved by introducing ultralow loadings of boron nitride nanotubes (BNNTs) into the nanocomposites to enhance sensitivity. Additionally, they utilized 3D printing during the fabrication process to create several micropatterned piezoelectric films, resulting in an enhanced piezoelectric output from the sensor (Zhang et al., 2020). Zeng et al. created a mask-image-projection-based stereolithography (MIP-SL) 3D-printed piezoelectric composite for ultrasonic devices, utilizing BaTiO3-based ceramics. These composites were designed with a honeycomb structure, taking advantage of the flexibility of 3D printing designs. The honeycomb-structured composites demonstrated a desirable piezoelectric constant of 60 pC/N and acceptable ferroelectric performance. Notably, the honeycomb structure sensitivity was twice as high as that of the solid brick structure (Zeng et al., 2020). Kim et al. modified piezoelectric nanoparticle-embedded polymer composites for 3D printing of microstructures suitable for sensor fabrication. They incorporated piezoelectric nanoparticles like BaTiO3 and BTO into photoliable polymer solutions, such as polyethylene glycol diacrylate. The researchers investigated the voltage output and piezoelectric coefficient of each combination. For the demonstration, they employed digital projection printing (DPP) technology to 3D print various microstructures, including dot arrays, square arrays, honeycomb arrays, mushroom-like arrays, cross arrays, and tapered cantilever arrays. These microstructures held the potential for sensor applications (Kim et al., 2014). Cui et al. engineered a 3D printing system that can manipulate the piezoelectric properties of materials. They created customized functional piezoelectric metamaterials, which were then employed to construct microstructures using 3D printing technology. By controlling parameters within the 3D printing system, they could precisely adjust the piezoelectric coefficient of the materials, providing a high degree of tunability for their piezoelectric properties (Cui et al., 2019).

In piezoelectric sensors, the electric poling process is considered crucial for fabrication. Proper alignment of electric dipoles is essential to fully exploit the benefits of piezoelectric sensors. Applying an electric field aid in arranging these dipoles, enhancing the sensors’ piezoelectric output. However, the traditionally cumbersome electric poling process has presented a challenge when incorporating 3D printing into piezoelectric sensor production. To address this challenge, researchers have undertaken efforts to combine 3D printing technologies with the electric poling process. This integration has streamlined the fabrication process, significantly reducing the time and cost associated with manufacturing piezoelectric sensors. Bodkhe et al. introduced an innovative fabrication method combining 3D printing with electrical poling process. This technique was applied to polyvinylidene fluoride (PVDF) nanocomposites containing 10wt% barium titanate (BaTiO3) nanoparticles, concurrently conducting 3D printing and electrical poling with an electric field of 1 MV/m. This technology yielded a remarkable threefold increase in specific charge output compared to unpoled PVDF. Furthermore, the researchers showcased the capability to create conformal sensors using this technology (Figure 5B) (Bodkhe et al., 2018). Kim et al. integrated Fused Deposition Modeling (FDM) 3D printing technology with the corona poling process, a type of electric poling. This combination involved using high electric fields in the corona poling process to fabricate PVDF films through FDM 3D printing. This novel approach, referred to as the 'Integrated 3D Printing and Corona Poling Process’ (IPC), significantly improving the piezoelectric current output, achieving a level of up to ±0.106 nA when applied with 12 kV electric voltage (Kim H. et al., 2017). Fuh et al. introduced wavy-substrate self-powered sensors (WSS) utilizing 3D printing technology and an *in situ* poling method. The sensor substrate was 3D printed with a wavy surface design, such as a square or sinusoidal surface. This 3D-printed structure enhanced the sensor’s sensitivity and increased its output. To complete the sensor, the substrate was coated using the near-field electrospinning (NFES) technique and underwent an *in situ* poling process simultaneously (Fuh et al., 2017). Košir et al. achieved the integration of fused filament fabrication (FFF) 3D printing with electric poling to create a single process for fabricating piezoelectric sensors. This comprehensive fabrication process encompassed both electrode deposition and electrode poling. They utilized a commercial conductive filament to produce the electrodes, while a PVDF filament was employed to create the piezoelectric layer. The resulting film was subjected to an electric field of 16.5 MV/m. Two sensors were successfully manufactured, yielding in-plane sensitivities of 17.2 pC/n and 11.8 pC/N, respectively (Košir and Slavič, 2022).

### 3.4 Triboelectric sensor

Triboelectric sensors, or triboelectric nanogenerators (TENG), operate on repeated contact and separation of two materials with differing triboelectric properties. Driven by external mechanical stimulations, like human motion, these materials cyclically engage and disengage, generating an electrical potential. Such a mechanism can be used as a sensor or an energy-harvesting device within wearable technology (Song et al., 2020). Triboelectric sensors are advantageous in self-powering applications without external power sources.

Haque et al. created a triboelectric touch sensor through 3D printing technology to seamlessly integrate the sensor with 3D objects. They employed TangoBlack and PDMS as the 3D-printed materials to serve as the functional electrode layer in the triboelectric touch sensor. Additionally, the spacer, responsible for maintaining separation between the two electrodes, was crafted using 3D-printed polyamide (PA). The fabricated sensor exhibited a self-powering capability. It generated energy from tactile stimuli, eliminating the need for an external power source (Haque et al., 2018). Chen et al. employed additive manufacturing technique known as UV 3D printing. They successfully produced a flexible triboelectric nanogenerator (TENG) by combining ink extrusion with a UV curing system. This 3D printing process, utilizing photopolymer resin, created a highly precise, layer-by-layer structure for the triboelectrification layer. In this study, the ionic hydrogel was utilized as the electrode material. The resulting TENG demonstrated its performance across various applications, encompassing a self-powered SOS and distress signaling system, intelligent illumination footwear, and self-sustaining portable setups for temperature sensing and smartwatches (Chen et al., 2018). Qian et al. adopted the direct ink writing (DIW) 3D printing technique, allowing for the precise deposition of silver ink, cellulose nanofiber (CNF) ink, and polydimethylsiloxane (PDMS) to construct a 3D micro/nano-hierarchical pattern structure. As a result, researchers created an all-printed triboelectric nanogenerator (AP-TENG) that exhibited efficiency to drive 88 light emitting diodes. Notably, the device achieved a 175% increase in voltage output compared to a traditional TENG produced through conventional molding methods. This AP-TENG functioned as a self-powered sensor, adept at monitoring the movement of fingers and limbs (Qian et al., 2019). Huang et al. created the self-recovering triboelectric nanogenerator (TENG) using of 4D printing technology. The self-recovery capability is attributed to using shape-memory polymer, ensuring exceptional device performance and structural robustness even after thermal treatment. The printed device is able to capture mechanical energy, delivering a maximum output power density of 56 mW/m^2^. Additionally, it serves as a self-powered sensor, proficient in detecting the bending angles of human joints (Huang et al., 2021). Yi et al. harnessed the unique electronegative and conductive properties of MXene to design a self-powered sensing system by fabricating an MXene film using 3D extrusion printing and printable ink with adjustable viscosity through controlling the water content. This system incorporates a triboelectric nanogenerator (TENG), enabling it to operate independently without dependence on an external power source. The self-powered physiological sensing system delivered a power output of approximately 816.6 mW/m^2^, a sensitivity of around 6.03 kPa⁻^1^ with a minimum detection limit of ∼9 Pa, and a rapid response time of roughly 80 m. The resulting system is well-suited for real-time monitoring of physiological vital signs (Yi et al., 2022). Wang et al. employed a freeze-drying assisted 3D printing technique to create a deep-trap hierarchical structure using cellulose nanofiber (CNF) and MXene. This structural design was tailored explicitly for non-contact triboelectric nanogenerators and proved highly effective in sensing motion in various directions. The exceptional sensing capabilities were attributed to the increased presence of open pores and enhanced triboelectric surfaces within the structure (Wang et al., 2023).

## 4 Application

In biomedical research and healthcare, biosensors have emerged as indispensable tools for measuring and monitoring various physiological signals. These biosensors play a pivotal role in capturing the intricate details of bodily functions to provide real-time insights into health conditions. The choice of measurement site and the type of biosensor used will largely depend on the frequency band under consideration (Srivastava et al., 2017). This categorization has led to a classification framework differentiating signals into three main frequency units: high (50–3,000 Hz), mid (0–300 Hz), and low (0–10 Hz). Such a framework not only aids in comprehending signal processing intricacies but also enhances the selection and design of appropriate biosensing technologies.

### 4.1 High frequency

**FIGURE 6:**
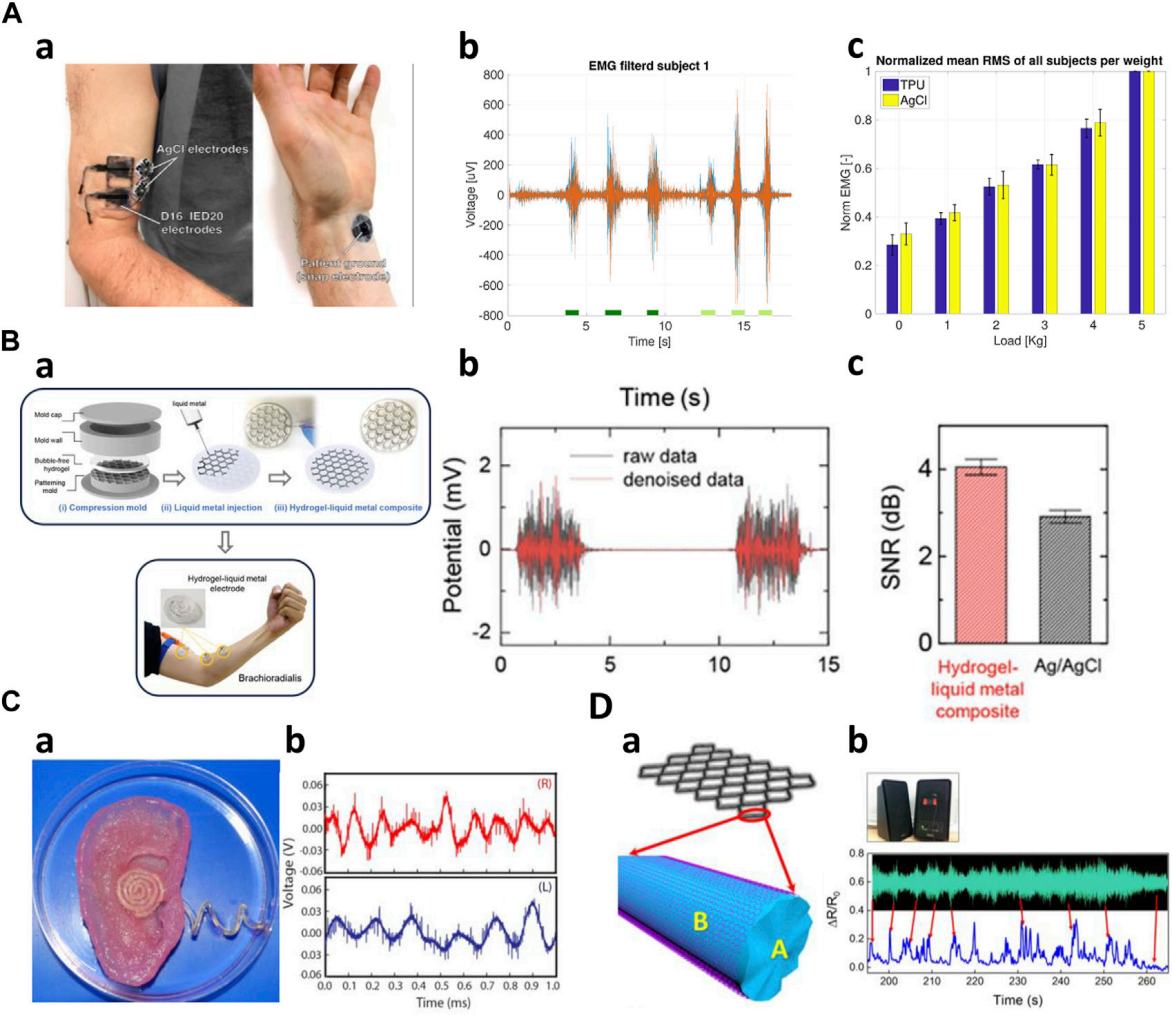
High frequencies application of 3D-printed kinematic biosensors **(A)** EMG measurement of 3D printed sensors (Wolterink et al., 2020). a) TPU electrodes and AgCl electrodes placed on a subject's biceps and wrist. b) Time-domain data measuring isometric and concentric contractions. c) Performance comparison of TPU and AgCl electrodes. **(B)** Creating sensors using 3D printed molds (Choi et al., 2020). a) A view of the hydrogel-liquid metal composite fabricated and attached to the arm. b) Measured raw data and denoise data. c) Comparing the performance of the composite and AgCl sensors. **(C)** AgNP-infused silicone electrodes printed on the bionic ear (Mannoor et al., 2013). a) A photo of the fabricated sensor's shape. b) Audio signal reception data. **(D)** The structure model of G-PDMS/RGO sensor (Ma et al., 2019). a) A schematic of the sensor. b) Measured sound wave profile of the music.

First of all, Electromyography (EMG) signals, operating within 50–3,000 Hz, represent some of the highest-frequency bioelectric signals in the human body. EMG sensing structures via 3D printing are utilized to fabricate custom sensors for integration into hearing aids, orthotics, and medical devices. Wolterink et al. fabricated EMG measurement electrodes by printing flexible thermoplastic polyurethane (TPU) doped with carbon black (Figure 6A). The electrode attached to the subject’s right biceps demonstrated comparable performance to an AgCl electrode attached to a similar location (Wolterink et al., 2020). Similarly, Schouten et al. fabricated electromyography electrodes with the carbon black added TPU (PI-ETPU) and stretchable silver ink (Engineered Conductive Materials). The authors compared the performance of the two 3D-printed electrodes. After subjects performed five isometric contractions, the electrodes made of silver ink were able to obtain more stable impedance measurements than the other controls (Schouten et al., 2022). Abass et al. printed rigid conductive PLA into small, perforated shapes to create dry electrodes. The electrodes were used to classify five different gestures through an EMG wearable device mounted on the subject’s right arm, so that the results were compared to actual gesture data. The testing results yielded a top classification accuracy of 85% on average (Abass et al., 2019). Choi et al. fabricated an electrode with the electrical pathway inside by injecting liquid metal into a 3D printed hydrogel mold (Figure 6B). The electrode performs almost identically to its off-state (initial state) resistance even after cutting the electrode due to the self-healing properties of the hydrogel. The fabricated electrode was attached to the biceps of a subject’s right arm to measure the EMG signals generated during an isotonic contraction, showing the similar but larger data peaks compared to the conventional Ag/AgCl electrode. The results confirms that the proposed electrodes can produce the reliable biological signals compared to the conventional electrodes (Choi et al., 2020). Additionally, researchers have yielded innovations in prosthesis, thereby expanding their utilization possibilities. Lee et al. created a prosthetic device by incorporating a 3D-printed socket and a capacitive pressure sensor. The sensor is composed of a polyurethane (TPU) substrate with conductive polylactic acid-carbon black compound (PLA-CB) electrodes sandwiching a dielectric made from Ecoflex 00–30. The printing process significantly improved the stability and sensitivity of the capacitive sensor. Using a prosthetic device with sensors, they suggested that different degrees of freedom could be achieved by measuring pressure changes and EMG signals through postural changes in the residual limb of an amputee (Lee et al., 2021).

Secondly, the biosensors primarily measure sound signals within the frequency range of 20 Hz–2000 Hz, falling within the human audible range, representing high-frequency ranges such as EMG signals. Using 3D printing technology, Mannoor et al. fabricated an artificial bionic ear with an antenna using a hydrogel matrix infused with cells in the anatomical shape of a human ear by incorporating a conductive polymer derived from silver nanoparticles (Figure 6C). This structure promotes the growth of cartilage *in vitro* around an induction coil antenna within the artificial ear. The antenna provided a frequency spectrum that demonstrated that a larger range of frequencies (1 MHz–5 GHz) than the audible signal frequency could be measured (Mannoor et al., 2013). In addition, Ma et al. constructed a highly stretchable G-PDMS/RGO composite open mesh by GO-coating a graphene-PDMS mixture (G-PDMS) (Figure 6D). The mesh utilizes a self-compensating double-aligned structure, where the resistance increases as the strain of the mesh increases, resulting in much higher sensitivity. The fabricated sensor exhibited a near-synchronous response to the acoustic wave profile of musical audio and was able to retain most of the characteristic peaks (Ma et al., 2019).

### 4.2 Mid frequency

Among various biological signals, the signals that fall in the relatively low frequency range of 0–300 Hz but having a continuous and regular cycle, are categorized as mid-frequency. Representative examples of the mid-frequency biological signals include the heart rate (pulse), and blood pressure. These biological signals are typically quantified by detecting the changes in electrical conductivity and electrical resistance in the stretchable sensor.

**FIGURE 7:**
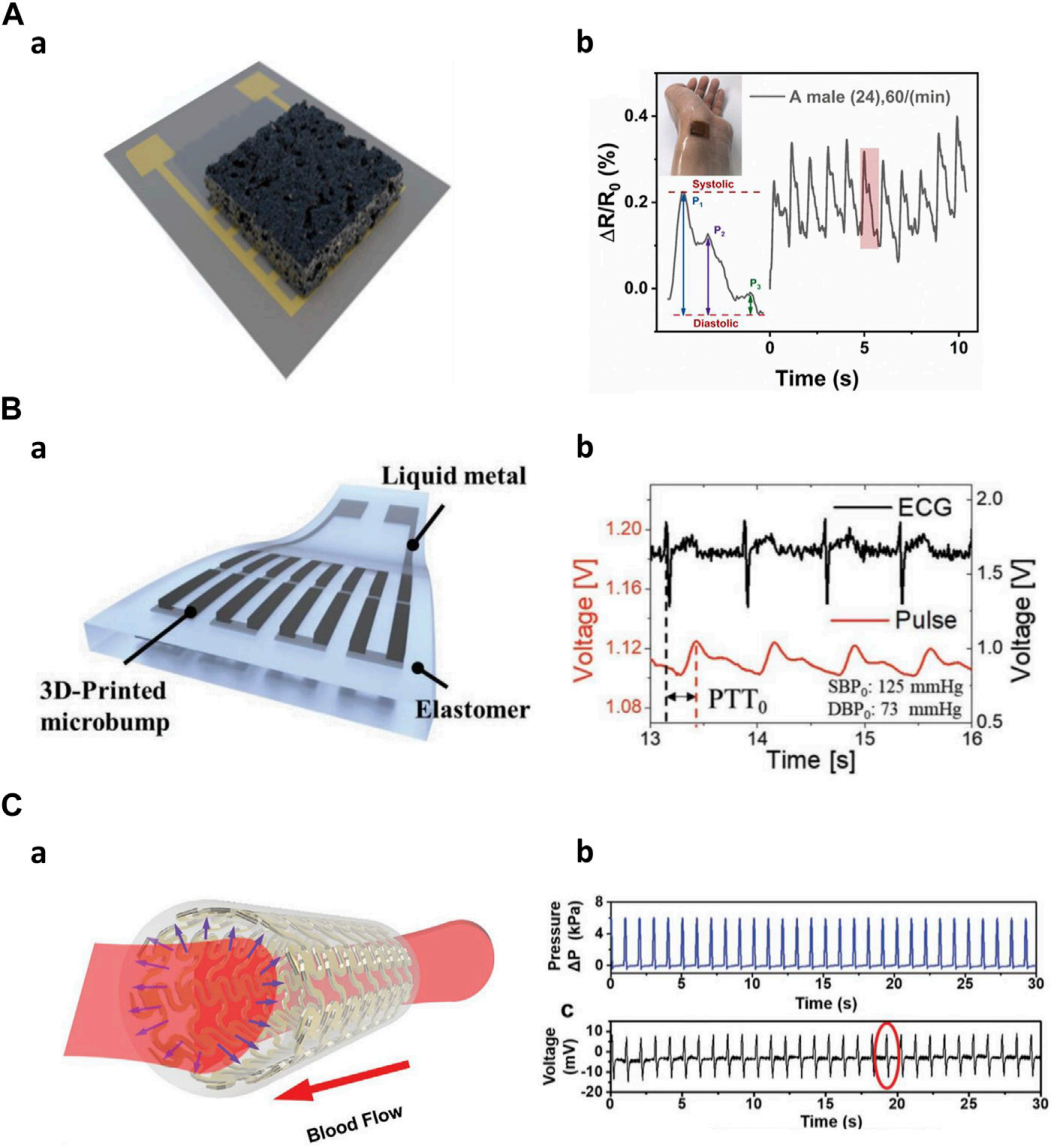
Mid frequencies application of 3D-printed kinematic biosensors **(A)** The nest-architecture-based wide-range pressure sensor (Guan et al., 2020). a) Schematic of the sensor. b) Real-time radial artery pulse monitoring. **(B)** 3D-printed rigid microbump-integrated liquid metal-based pressure sensor (Kim et al., 2019). a) Schematic of the sensor. b) Calculate Continuous epidermal pulse and ECG signals for PTT. **(C)** The artificial artery in response to blood pressure (Li et al., 2020). a) Schematic of the artificial artery. b) Real-time pressure change data inside the artery.

First, a sensor for measuring heart rate (pulse) can be fabricated using ionogel. Zhang et al. used ionogels to fabricate sensors with ultra-high elasticity and durability, and self-healing performance under UV irradiation at room temperature. The ionogel sensor can exhibit short response time and high sensitivity under different strain rates and pressures. By attaching the sensor to the subject’s wrist, the results showed that the pulse frequency was 71 times per minute, demonstrating that the measured radial pulse was very clear and stable (Zhang et al., 2022). Wei et al. fabricated an embedded 3D printed sensor based on a novel thermosetting printing ink by mixing Ecoflex 00–30 with carbon nanoparticles. The proposed pressure sensor was able to monitor Cun, Guan, and Chi, which are arterial pulses in traditional Chinese medicine, proving that it can be used in a smart arterial pulse diagnostic device (Wei et al., 2019). Inspired by ant nest design, the unique 3D multi-layer porous structure of the sensor achieves high sensitivity, low detection limit, and wide measurement range (Figure 7A). Attached to the subject’s left wrist, the sensor captures subtle dynamic information, allowing for real-time monitoring of the radial artery pressure pulse (Guan et al., 2020). Wu et al. created a piezoelectric sensor using a hybrid 3D printing approach that combined inkjet printing and extrusion printing methods. The sensor can accurately read current signals with a periodicity of 73 cycles per minute when attached to a subject’s wrist (Wu et al., 2020). A flexible stress sensor was developed through a 3D digital printing method based on PDMS and graphene nanosheets. The sensor shows high reliability and fast response time for pulse measurement (Shi et al., 2019). Guo et al. made a resistive tactile sensor with a complex structure including grid-structured electrodes and a coil-shaped sensor layer. The sensor was attached to the wrist and by measuring the pulse, monitored the sedentary state and post-exercise state (Guo et al., 2017).

Second, various biosensors can measure blood pressure using their ability to detect pressure changes. 3D-printed rigid microbump-integrated liquid metal-based soft pressure sensor (3D-BLiPS) detect pressure changes through the integration of 3D-printed rigid microbump arrays and liquid metal microchannels (Figure 7B). Blood pressure was measured using the pulse transit time (PTT) method, which utilizes the time difference between two different pulse measurements. This method allows for continuous pulse monitoring and a non-invasive blood pressure monitoring system (Kim et al., 2019). A wireless pressure sensor was integrated into a 3D-printed biocompatible and biodegradable polymeric stent. The sensor combined with the stent morphology creates an artificial artery system that exhibits relatively high sensitivity to the normal human blood pressure range and can be effectively used for real-time blood pressure monitoring (Figure 7C) (Park et al., 2019; Li et al., 2020).

### 4.3 Low frequency

Kinematic bio-signals with frequencies below 10 Hz, such as gait, joint movements, swallowing, respiration, and hand gestures, fall into the category of low-frequency data. Such low-frequency kinematic bio-signals are significant as they capture essential variations in human movements, making them critical for biomedical applications and health monitoring applications.

**FIGURE 8:**
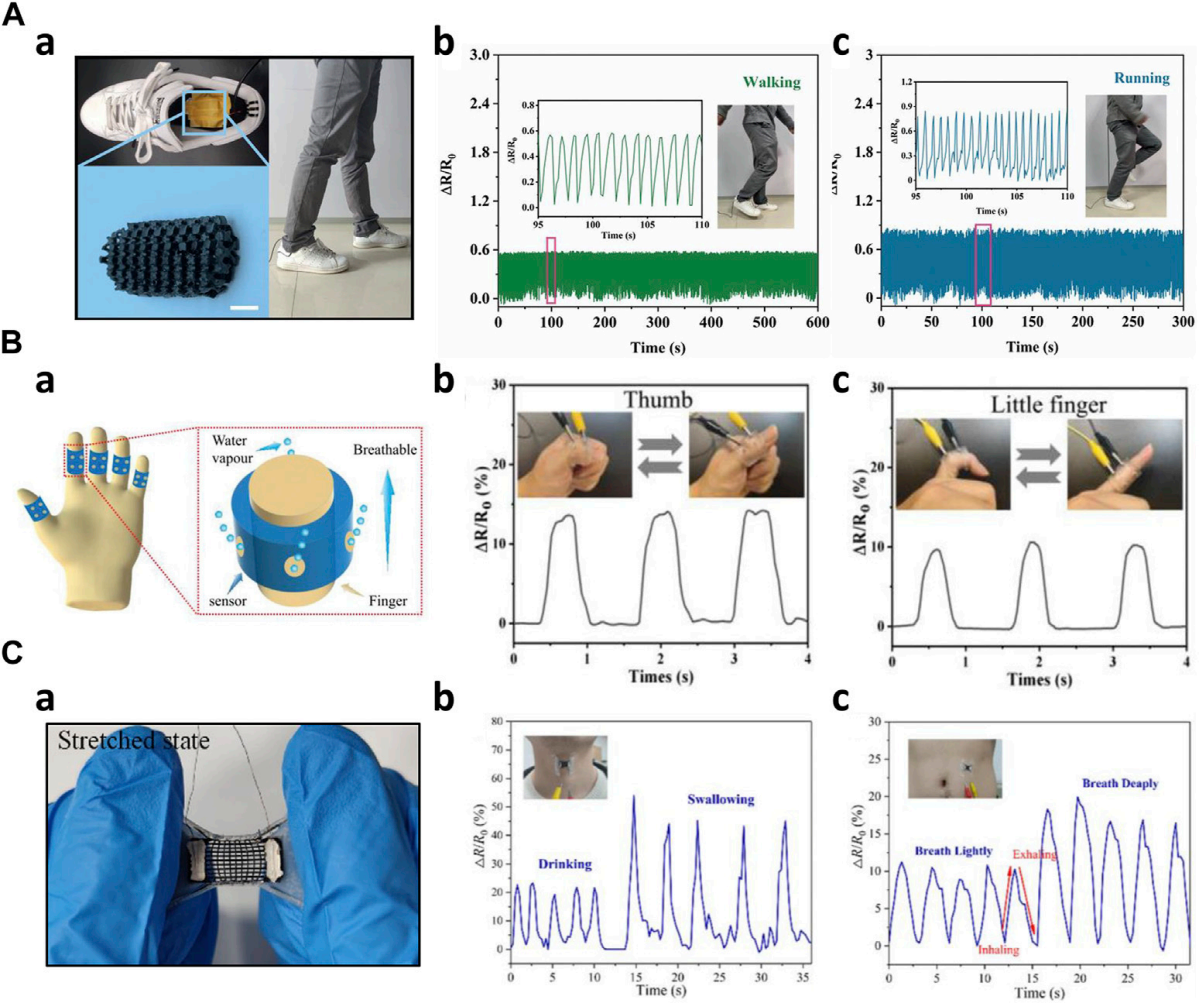
Low frequencies applications of 3D-printed kinematic biosensors **(A)** Walking and running Monitoring a) 3D printed sensor embedded into the shoe insole (Peng et al., 2021) b) Monitoring of the human walking c) Monitoring of the human running **(B)** 3D printed wearable and breathable strain sensor (Zhou et al., 2019) a) Schematic image of the strain sensor b) Thumb joint movement monitoring c) Little finger joint movement monitoring **(C)** Auxetic bilayer strain sensor (Wang et al., 2021) a) Image of the strain sensor in stretched state b) Drinking and swallowing monitoring c) Respiration monitoring.

First, 3D-printed biosensors are incorporated into the shoe to monitor kinematic gait signals such as walking, running, or pressure monitoring. Moreover, the data provides important information that can be used in disease monitoring. Oprel et al. developed a capacitive shear sensor using an innovative printing technique. This sensor was designed for potential applications in detecting diabetes by embedding the sensor in a shoe insole to monitor shear stress at the feet (Oprel et al., 2023). Peng et al. fabricated a porous flexible strain sensor (PFSS) by casting polyurethane/carbon nanotubes composites into 3D printed sacrificial molds. PFSS can also be used in pressure sensing. The work demonstrated the PFSS as a wearable device for human motion monitoring. They integrated the PFSS into a shoe and classified human motions such as running and walking (Figure 8A) (Peng et al., 2021). Kim et al. created a resistive pressure sensor using a porous structured metamaterial for stance and motion analysis. The Young’s modulus of these sensors was adjustable by varying the density of the porous structure. By incorporating eight pressure sensors into a sandal, the system could identify different gait postures, including heel strike, foot flat, mid stance, heel rise, and toe-off. This sensor setup allowed for a comprehensive analysis of various aspects of gait and posture (Kim et al., 2023). Kim et al. manufactured a multi-axis pressure sensor utilizing Fused Filament Fabrication (FFF) 3D printing technology. This pressure sensor featured a temperature sensing element and could detect various kinematic changes in human movements. When integrated into a flip-flop design, the sensor effectively distinguished different gait motions, including stomping, jumping, dragging forward, and dragging backward (Kim et al., 2021). Bodkhe et al. created a piezoelectric sensor by simultaneously utilizing 3D printing and electric poling techniques, significantly improving its sensitivity. These sensors were placed on the front and back of a shoe, effectively distinguishing between different gait motions, such as walking and stamping (Bodkhe et al., 2018).

Secondly, biosensors were employed to monitor joint movements in various human joints. These basic strain sensors provide essential information about movements induced by humans, serving as a fundamental component in the monitoring of joint activity. Loh et al. manufactured a metamaterial capacitive sensing array capable of conforming to curved joints, such as elbows and knees. This auxetic-shaped sensor array was wrapped around the elbow and functioned as a force sensor. As a person bent their arm, the capacitance changed, and the 6 × 6 sensing arrays detected each variation in capacitance. The collected data was then decomposed and analyzed to measure force distribution (Loh et al., 2021). Guo et al. designed an organohydrogel-based breathable strain sensor. This sensor was applied to each of the five fingers and used to monitor changes in resistance as the finger joints were bent. The mechanical properties of the hydrogels provided wearability and breathability while enabling precise monitoring of joint movements (Figure 8B) (Guo et al., 2023). Zhao et al. created a flexible strain sensor using the previously developed embedded 3D printing (e−3DP) technology (Muth et al., 2014). They applied this sensor to the wrist, knee, and finger, respectively, to monitor changes in resistance corresponding to the angle variations as the joints were tilted from 0 to 60° (Zhao C. et al., 2020).

Thirdly, 3D-printed biosensors were employed to monitor subtle biosignals induced by motor functions such as voice, swallowing motions, respiration, phonation, and blinking. These sensors play an instrumental role in tracking and analyzing the physiological activities attached to specific body parts. Wang et al. designed stretchable strain sensors with an auxetic bilayer structure. These sensors were created by combining DIW printing and ink spraying techniques. In a practical demonstration, these strain sensors could detect and monitor activities such as swallowing, drinking, and respiration (Figure 8C) (Wang et al., 2021). Xia et al. introduced a novel 3D-printed flexible piezoresistive sensor with a range of microstructures, resulting in substantial improvements in sensor performance. When applied to a 23-year-old male, the sensor was attached to the neck and successfully detected human vocalizations such as 'Hello’ and 'Hi’ along with cheek bulging. Its high sensitivity in low-pressure ranges produced valid output signals. Moreover, the sensor effectively monitored cheek bulging when affixed to the individual’s cheek (Xia et al., 2021). Zhu et al. developed a piezoresistive pressure sensor inspired by human skin, with high sensitivity and a broad sensing range. This sensor demonstrated impressive capabilities of detecting faint signals, including gripping an apple and recognizing vocal signals. Notably, it could also serve as a tool for disease prevention by being worn as a sleeve sensor on a limb, allowing for the monitoring of thrombosis in the limb (Zhu G. et al., 2022). Wang et al. developed a hierarchical porous structure with a multi-modulus architecture piezoresistive sensor, which was employed as a wearable sensor to monitor swallowing and blinking motions. Additionally, this sensor effectively captured phonation, including phrases such as 'Hello!' 'How are you?' 'How old are you?' and 'Nice to meet you.’ (Wang et al., 2019).

## 5 Conclusion

Additive manufacturing technology has made significant contributions to the field of biosensors in the context of wearable devices and artificial skins. Innovative printing techniques have emerged to enhance sensor performance and durability while simultaneously reducing production costs. Moreover, the development of additive manufacturing technology has enabled the creation of intricate microstructures, amplifying the sensitivity and sensing range of biosensors. This advantage has led to a growing adoption of additive manufacturing in biosensor fabrication, establishing it as a prevailing trend in the field.

However, despite the numerous merits of additive manufacturing, there are still challenges that need to be addressed. So far, there is a limitation in the availability of biocompatible and printable materials that are well suited for biosensor fabrication. The printable materials with multi-functionality enhance the applicability of 3D printed biosensors. In terms of multi-functionality, one of the recent advancements in additive manufacturing is 4D printing. 4D printing represents an evolved form of 3D printing, introducing the dimension of time as its defining feature. The ‘fourth dimension’ alludes to the element of time. 4D printing stands as a progression from 3D printing, endowing products with the capability to adapt their shape, structure, or functionality over time. This achievement is made possible through the utilization of smart materials or substrates that exhibit dynamic responses to time or specific stimuli, thereby enabling physical transformations or structural adjustments in products (Khalid et al., 2022). Utilization of 4D printing technology in the smart skin patch might enhance user convenience and sensor efficiency, facilitating the monitoring of the user’s health status and the tracking of environmental conditions.

Furthermore, additive manufacturing has yet to fully encompass the entire biosensor fabrication process and it is only partially integrated into sensor production. While ongoing efforts are aimed at expanding the scope of additive manufacturing by incorporating more features, these challenges continue to impede the widespread adoption of AM technology in biosensor development.

The future of wearable electronic devices hinges on soft and flexible sensors. Unlike the current rigid and cumbersome commercial wearable devices, soft, biocompatible, and conformal devices are poised to become the mainstream. The inherent advantages of soft devices, particularly their superior performance in biosensing and healthcare applications, make the transition to skin-conformal soft biosensors an inevitable revolution. Additively manufactured, skin-attachable biosensors will continually advance their performance, thanks to ongoing developments in material science and novel manufacturing technology. Novel materials will play a pivotal role in creating robust, high-performance sensors with excellent printability.

As the existing limitations of soft wearable devices, including challenges related to durability, power supply, washability, and biocompatibility, are progressively addressed and resolved, these devices can catalyze advancements in biosensors and additive manufacturing technology. Envisioning the future, we anticipate a scenario where the complete biosensor fabrication process seamlessly integrates with a 3D printing platform. An automated printing device would efficiently manage every manufacturing stage, from material preparation to printing and arrangement. This transformative approach promises to usher in an era characterized by low-cost, rapid mass production, paving the way for potential large-scale commercialization.
