# Corrigendum: Recent advances of additively manufactured noninvasive kinematic biosensors

**DOI:** 10.3389/fbioe.2025.1560319

**Published:** 2025-02-14

**Authors:** Jeonghoon Lee, Sangmin Park, Jaehoon Lee, Namjung Kim, Min Ku Kim

**Affiliations:** ^1^ Department of Mechanical Convergence Engineering, Hanyang University, Seoul, Republic of Korea; ^2^ Department of Mechanical Engineering, Gachon University, Seongnam, Republic of Korea; ^3^ School of Mechanical Engineering, Hanyang University, Seoul, Republic of Korea

**Keywords:** noninvasive biosensors, additive manufacturing, 3D printing, kinematic sensors, low-cost fabrication

In the published article, there was an error in the **Funding statement**. “The funder Hanyang University (HY-202100000003082) was not included.” The correct Funding statement appears below.

The authors apologize for this error and state that this does not change the scientific conclusions of the article in any way. The original article has been updated.

